# Robust In-Flight Sensor Fault Diagnostics for Aircraft Engine Based on Sliding Mode Observers

**DOI:** 10.3390/s17040835

**Published:** 2017-04-11

**Authors:** Xiaodong Chang, Jinquan Huang, Feng Lu

**Affiliations:** Jiangsu Province Key Laboratory of Aerospace Power System, Nanjing University of Aeronautics and Astronautics, Nanjing 210016, China; cxd18762406179@163.com (X.C.); lufengnuaa@126.com (F.L.)

**Keywords:** commercial aircraft engine, health degradation, sensor fault diagnostics, sliding mode observer

## Abstract

For a sensor fault diagnostic system of aircraft engines, the health performance degradation is an inevitable interference that cannot be neglected. To address this issue, this paper investigates an integrated on-line sensor fault diagnostic scheme for a commercial aircraft engine based on a sliding mode observer (SMO). In this approach, one sliding mode observer is designed for engine health performance tracking, and another for sensor fault reconstruction. Both observers are employed in in-flight applications. The results of the former SMO are analyzed for post-flight updating the baseline model of the latter. This idea is practical and feasible since the updating process does not require the algorithm to be regulated or redesigned, so that ground-based intervention is avoided, and the update process is implemented in an economical and efficient way. With this setup, the robustness of the proposed scheme to the health degradation is much enhanced and the latter SMO is able to fulfill sensor fault reconstruction over the course of the engine life. The proposed sensor fault diagnostic system is applied to a nonlinear simulation of a commercial aircraft engine, and its effectiveness is evaluated in several fault scenarios.

## 1. Introduction

Sensors in commercial aircraft engines operate in severe hostile conditions, thus they are prone to faults and failures. Any undetected sensor faults may cause disastrous consequences to engine control loops, and even threaten flight safety. Therefore, the design of the on-board sensor fault detection and isolation (FDI) system is critical to enhance the engine’s reliability, efficiency, and safety during flight. With the increasing complexity and intelligence of engine control logics, sensor fault diagnostics tend to take on more tasks. Apart from the fault detection and isolation, the “fault reconstruction” is a more advanced technique in which faults are identified with exact shape and magnitude, such that more precise maintenance work can be done or active fault-tolerant control schemes can be applied. Another challenging issue for establishing an in-flight sensor FDI system is the robustness against engine performance degradation. As parts wear from regular use, aircraft engines will exhibit gradual degradation in rotating components over their operating life. The degradation will cause sensed measurements to deviate from the nominal value, which may eventually lead to misdiagnosis in sensor FDI. Therefore, a reliable sensor fault diagnostic scheme is expected to accurately detect and identify the fault, while to be robust enough to engine degradations.

In general, most FDI-related methods can be split into data-based and model-based methods. The latter one, utilizing all model information available, tends to be more accurate in diagnosis without a priori knowledge of faults. A well-known model-based method for the in-flight sensor FDI of aircraft engines is initially investigated by Merrill et al. [1], who utilized a bank of Kalman filters (KF) to detect and isolate sensor faults. Then the work was extended by Kobayashi et al. [2] to augment the FDI system with detecting actuator and component faults by applying more Kalman filters. The main concept of their work is that each Kalman filter is designed to be related to one specific fault, and then residuals from each Kalman filter will be compared to a set threshold to determine whether there is a fault in the corresponding channel. However, this method is hard to handle concurrent faults. To address the degradation problem, Simon [3] and Armstrong et al. [4] described the enhanced sensor FDI scheme by updating the health baseline model used for sensor diagnosis periodically. The architecture contains real-time adaptive performance model (RTAPM) to estimate health condition, and the performance baseline model (PBM) updated by health estimation results off-board to detect faults. The shortcoming is that the Kalman filters need to be redesigned once the PBM is updated. Thus, the updating period is decided by weighting the pros and cons of the diagnostic accuracy and the operating costs. To address this problem, Kobayashi et al. [5] improved the on-line sensor diagnostics by using Hybrid Kalman Filter (HKF), which lends itself to the health baseline update.

Sliding mode observer techniques have gained much attention in fault diagnosis recently, due to their remarkable capabilities of fault reconstruction. The nonlinear discontinuous term can be designed to maintain a sliding motion, while faults can be reconstructed by analyzing the so-called “equivalent output estimation error injection” term, instead of being just isolated by analyzing residuals in KF-bank-based methods. In the work of Tan et al. [6] and Alwi et al. [7], the robust sliding mode observers were applied to reconstruct sensor faults for an aero-engine with uncertainties involved. Rahme et al. [8] investigated a SMO-based sensor fault diagnosis scheme for a gas turbine under degraded component scenarios. In [8], the observer was proved to maintain stability with degradation injection, but the reconstruction of sensor faults was polluted. So far, uses of SMO in fault detection have mainly been in handling actuator/sensor fault cases. In our previous research [9], the feasibility and potential of sliding mode observer to be applied to an aircraft engine health monitoring system was investigated.

In this paper, an integrated sensor fault diagnostic scheme is proposed based on sliding mode observers. Two SMOs are employed in this architecture: one is for degraded performance tracking, and another for sensor fault reconstruction. Compared to KF-bank-based methods, the proposed scheme is structurally simplified, and faults can be not only located, but a direct estimate of the size of the fault is also obtained. In addition, concurrent sensor faults can be reconstructed by the described method as well. Similar to the basic architecture in [3,4], the current health condition after each flight is estimated by degraded performance tracking, which is used to update the baseline model for on-board sensor fault diagnosis in the next flight cycle. However in our scheme, the update operations and the whole diagnostic algorithms can be executed in on-board computers, with no ground-based effort required, thus the difficulties associated with off-board to on-board data transmission are avoided. With such an advantage, the degradation update process can be executed after each flight, which is of great importance to increasing diagnosis accuracy.

In the following sections of this paper, a brief description of the considered aircraft engine is firstly given. Next, a second-order sliding mode observer (SOSMO) designed to reconstruct in-flight sensor faults is described, followed by the same algorithm applied to build another SOSMO to fulfill on-board health performance tracking, and the overall architecture of the integrated sensor fault diagnostic system is depicted. Then, the performance of the proposed scheme is evaluated in a nonlinear simulation environment. Finally, conclusions are presented.

## 2. Aircraft Engine Description

**Figure 1:**
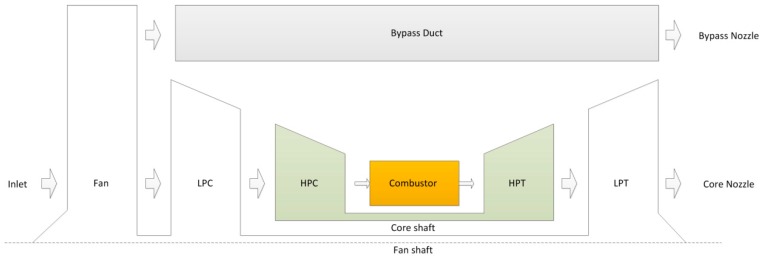
Schematic description of two-spool turbofan engine.

A two-spool turbofan engine is considered in this paper, of which the schematic model is shown in Figure 1. The airflow is supplied by a single inlet. Airflow passes through the fan and separates into two streams: one passes through engine core path, and the other passes through the bypass duct. Fuel is injected in the combustor and burned to produce the hot gas to drive the turbines. The fan and low pressure compressor (LPC) are driven by a low pressure turbine (LPT), while the high pressure compressor (HPC) is driven by a high pressure turbine (HPT). The airflow leaves through the nozzle. The notations used in this paper and their descriptions are shown in Table 1.

As engine parts wear from regular operation, the lifespan of components will be reduced. The aging of components is reflected in slow-evolving changes in internal flow capacities and component efficiencies, thus these capacities and efficiencies of components are chosen as “health parameters” to reflect component health conditions. The degradations of health parameters are described as

(1) $$\Delta h_{i}=h_{i}/h_{i,r}-1,\;\;\;i=1,..,8$$

where *h_i_* is the health parameter and *h_i,r_* denotes the nominal value of *h_i_*. A normalized health parameter varies between 0 and 1, with 1 representing a healthy component and 0 a “fully deteriorated” one. The maximum level of deterioration indicates an engine overhaul is necessary.

Mechanical system dynamics due to rotating inertias constitute the most important contribution to engine transient behavior. Thus, rotating dynamics are the most important dynamics to be considered. In view of this, the state vector ***x*** is chosen as ${\left[N_{L},N_{H}\right]}^{T}$. Newton’s law for rotating masses is applied to each shaft as

(2) $${\dot{N}}_{L}=f_{1}\left(N_{L},N_{H},u,h,v\right)\;{\dot{N}}_{H}=f_{2}\left(N_{L},N_{H},u,h,v\right),$$

where *f*_1_ and *f*_2_ are the net torques delivered by LPT and HPT. ***u*** is the control input *W*_f_, h is the health parameter vector and ***v*** denotes the external parameters (flight condition). The available measurements are defined by the standard suite of sensors found in the tactical turbofan engine control system. Then the output vector y is chosen as ${\left[N_{L},N_{H},T_{25},P_{25},T_{3},P_{3},T_{495}\right]}^{T}$. 

For developing a sensor fault diagnostic algorithm of aircraft engine, one challenge in achieving reliable results is the effect of degradation. Fault diagnostic algorithms are generally designed under a nominal healthy condition, which constitutes a reference baseline for diagnostics. However, the fact that both sensor faults and degradations cause measurement deviations may result in misdiagnosis in degrading engines. The idea here is to develop a health monitoring system to update the baseline model off-line, and then to maintain the effectiveness of sensor fault diagnostic system over the course of the engine life.

Given the available sensor suite constraints, observability studies indicate seven health parameters could be discerned properly. The statistical data analyses from the described engine indicate that $h_{8}$ deteriorates much less in the degrading process compared to other health parameters [9], thus by ignoring $h_{8}$, the health parameter vector to be monitored is chosen as ${\left[\Delta h_{1},\Delta h_{2},\Delta h_{3},\Delta h_{4},\Delta h_{5},\Delta h_{6},\Delta h_{7}\right]}^{T}$.

## 3. Sensor Fault Diagnostic

In this paper, the proposed sensor fault diagnostic system is constructed based on sliding mode observers. Two SMOs are involved in the scheme: one is for health degradation tracking and another for sensor fault diagnostic. This section describes the design of the observer for sensor fault reconstruction. Since most FDI-related work of commercial aircraft engines is concerned with the cruise state, a state variable model (SVM) representing the engine dynamic in a small range around a steady-state operating point is appropriate and convenient for the observer design. Considering h as artificial inputs, the SVM of the cruise operating point can be obtained

(3) $$\dot{x}\left(t\right)=Ax\left(t\right)+Bu\left(t\right)+Lh\left(t\right)\;\;y\left(t\right)=Cx\left(t\right)+Du\left(t\right)+Mh\left(t\right)+f\left(t\right),$$

where $A\in {\mathbb{R}}^{n\times n}$, $B\in {\mathbb{R}}^{n\times m}$, $C\in {\mathbb{R}}^{p\times n}$, $D\in {\mathbb{R}}^{p\times m}$, $L\in {\mathbb{R}}^{n\times q}$, and $M\in {\mathbb{R}}^{p\times q}$ are constant coefficient matrices. $f\left(t\right)\in {\mathbb{R}}^{p}$ denotes the sensor fault signal. Here, n=2, m=1, p=7, q=7. Assume f(t), h(t), and their first-time derivatives are unknown but bounded

(4) $$\|f\left(t\right)\|<\alpha_{1},\;\|\dot{f}\left(t\right)\|<\alpha_{2},\;\|h\left(t\right)\|<\beta_{1},\;\|\dot{h}\left(t\right)\|<\beta_{2},$$

where $\alpha_{1}$, $\alpha_{2}$, $\beta_{1}$, and $\beta_{2}$ are known real scalars. The notation ‖·‖ represents the Euclidean norm for vectors and the induced spectral norm for matrices.

A new state $z\left(t\right)\in {\mathbb{R}}^{p}$, which is a filtered version of y(t), is introduced

(5) $$\dot{z}\left(t\right)=-A_{f}z\left(t\right)+A_{f}y\left(t\right),$$

where $-A_{f}\in {\mathbb{R}}^{p\times p}$ is a stable filter matrix. Typically $A_{f}$ is in the form of a diagonal matrix with positive entries where the diagonal elements represent inverse time constants. Substituting z(t) for y(t) in Equation (3), and combining x(t) and z(t) to create an augmented state $x_{a}\left(t\right)\in {\mathbb{R}}^{n+p}$, the following representation can be obtained

(6) $$\left[\begin{array}{c} \dot{x}\left(t\right)\\ \dot{z}\left(t\right) \end{array}\right]=\underset{A_{a}}{\underset{︸}{\left[\begin{array}{cc} A & 0\\ A_{f}C & -A_{f} \end{array}\right]}}\underset{x_{a}\left(t\right)}{\underset{︸}{\left[\begin{array}{c} x\left(t\right)\\ z\left(t\right) \end{array}\right]}}+\underset{B_{a}}{\underset{︸}{\left[\begin{array}{c} B\\ A_{f}D \end{array}\right]}}u\left(t\right)+\underset{H_{a}}{\underset{︸}{\left[\begin{array}{c} L\\ A_{f}M \end{array}\right]}}h\left(t\right)+\underset{F_{a}}{\underset{︸}{\left[\begin{array}{c} 0\\ A_{f} \end{array}\right]}}f\left(t\right)\;z\left(t\right)=\underset{C_{a}}{\underset{︸}{\left[\begin{array}{cc} 0 & I_{p} \end{array}\right]}}\left[\begin{array}{c} x\left(t\right)\\ z\left(t\right) \end{array}\right],$$

where $A_{a}\in {\mathbb{R}}^{\left(n+p\right)\times \left(n+p\right)}$, $B_{a}\in {\mathbb{R}}^{\left(n+p\right)\times m}$, $H_{a}\in {\mathbb{R}}^{\left(n+p\right)\times q}$, $C_{a}\in {\mathbb{R}}^{p\times \left(n+p\right)}$, $F_{a}\in {\mathbb{R}}^{\left(n+p\right)\times p}$, and $I_{p}\in {\mathbb{R}}^{p\times p}$ denotes identity matrix.

For the system described in Equation (6), the aim is to design a second-order sliding mode observer to identify sensor faults via “fault reconstruction” technique. As argued in [10], the necessary and sufficient conditions for the existence of a stable sliding motion and feasibility of fault reconstruction are:

1. The first Markov parameter (the product of $C_{a}$ and $F_{a}$) must have full column rank;
2. Any invariant zeroes (if there exists) of $\left(A_{a},F_{a},C_{a}\right)$ are Hurwitz.

It is easy to verify $rank\left(C_{a}F_{a}\right)=rank\left(A_{f}\right)=p$, then by constructing the Rosenbrock matrix for $\left(A_{a},F_{a},C_{a}\right)$, the invariant zeros of $\left(A_{a},F_{a},C_{a}\right)$ are given by the values of s for which

(7) $$R_{a}\left(s\right)=\left[\begin{array}{ccc} sI_{n}-A & 0 & 0\\ -A_{f}C & sI_{p}+A_{f} & A_{f}\\ 0 & I_{p} & 0 \end{array}\right]<n+2p.$$

It is clear that

(8) $$rank\left(R_{a}\left(s\right)\right)=rank\left(sI_{n}-A\right)+rank\left(\left[\begin{array}{cc} I_{p} & 0\\ sI_{p}+A_{f} & A_{f} \end{array}\right]\right).$$

If s is not an eigenvalue of A, then $det\left(sI_{n}-A\right)\neq 0$, and $Rank\left(R_{a}\left(s\right)\right)=n+2p$. Hence, the invariant zeros of $\left(A_{a},F_{a},C_{a}\right)\in \lambda \left(A\right)$. Therefore, to ensure the invariant zeros of $\left(A_{a},F_{a},C_{a}\right)$ are Hurwitz, the open-loop system matrix A in Equation (3) is required to be stable, and this condition is inherently satisfied by engine natures.

Next, a two-order sliding mode observer is designed based on Equation (6). Define $e_{z}\left(t\right)=\hat{z}\left(t\right)-z\left(t\right)$ as output estimation error, where $\hat{z}\left(t\right)$ is the estimate value of z(t). The proposed SMO has the following structure

(9) $${\dot{\hat{x}}}_{a}\left(t\right)=A_{a}{\hat{x}}_{a}\left(t\right)+B_{a}u\left(t\right)-G_{l}e_{z}\left(t\right)+G_{n}\nu \left(t\right)\;\;\hat{z}\left(t\right)=C_{a}{\hat{x}}_{a}\left(t\right),$$

where ${\hat{x}}_{a}\left(t\right)$ is the estimate value of $x_{a}\left(t\right)$. $G_{l}\in {\mathbb{R}}^{\left(n+p\right)\times p}$, $G_{n}\in {\mathbb{R}}^{\left(n+p\right)\times p}$ are linear gain matrix and nonlinear gain matrix, respectively. Define $e_{z}\left(t\right)={\left[e_{z,1}\left(t\right),e_{z,2}\left(t\right),..,e_{z,p}\left(t\right)\right]}^{T}$, then $\nu \left(t\right)={\left[\nu_{1}\left(t\right),\nu_{2}\left(t\right),..,\nu_{p}\left(t\right)\right]}^{T}$ is defined component-wise as

(10) $$\nu_{i}\left(t\right)=-\psi sign\left(e_{z,i}\left(t\right)\right){\left|e_{z,i}\left(t\right)\right|}^{1/2}+d_{i}\left(t\right)\;{\dot{d}}_{i}\left(t\right)=-\varsigma sign\left(e_{z,i}\left(t\right)\right)-\varphi e_{z,i}\left(t\right)\;\;\;i=1,2,..,p,$$

where ψ, ς, and φ are design scalars to be chosen. Define $e\left(t\right)={\hat{x}}_{a}\left(t\right)-x_{a}\left(t\right)$ as state estimation error. The following error system is obtained from Equations (6) and (9)

(11) $$\dot{e}\left(t\right)=A_{a}e\left(t\right)-G_{l}e_{z}\left(t\right)+G_{n}\nu \left(t\right)-H_{a}h\left(t\right)-F_{a}f\left(t\right).$$

According to the form of $C_{a}$, e(t) can be partition as ${\left[e_{1}^{T}\left(t\right),e_{z}^{T}\left(t\right)\right]}^{T}$ where $e_{1}\left(t\right)\in {\mathbb{R}}^{n}$. Let $G_{n}=\left[\begin{array}{c} 0\\ I_{p} \end{array}\right]$ and $G_{l}=\left[\begin{array}{c} 0\\ -A_{f}+\chi I_{p} \end{array}\right]\;$where χ is a real scalar to be chosen, then the error system can be written as

(12) $$\left[\begin{array}{c} {\dot{e}}_{1}\left(t\right)\\ {\dot{e}}_{z}\left(t\right) \end{array}\right]=\left[\begin{array}{cc} A & 0\\ A_{f}C & -\chi I_{\tilde{p}} \end{array}\right]\left[\begin{array}{c} e_{1}\left(t\right)\\ e_{z}\left(t\right) \end{array}\right]+\left[\begin{array}{c} 0\\ I_{p} \end{array}\right]\nu \left(t\right)-\left[\begin{array}{c} L\\ A_{f}M \end{array}\right]h\left(t\right)-\left[\begin{array}{c} 0\\ A_{f} \end{array}\right]f\left(t\right).$$

The objective is to force the output error $e_{z}\left(t\right)$ to zero in finite time and induce a sliding mode on the sliding manifold

(13) $$S=\left\{{\left[\begin{array}{cc} e_{1}^{T}\left(t\right) & e_{z}^{T}\left(t\right) \end{array}\right]}^{T}|e_{z}\left(t\right)=0\right\}.$$

Considering the structure of ν(t) in Equation (10) and substituting Equation (10) into Equation (12), the equation related to $e_{z}\left(t\right)$ in Equation (12) can be written component-wise as

(14) $${\dot{e}}_{z,i}\left(t\right)=-\psi sign\left(e_{z,i}\left(t\right)\right){\left|e_{z,i}\left(t\right)\right|}^{1/2}-\chi e_{z,i}\left(t\right)+{\left(A_{f}C\right)}_{i}e_{1}\left(t\right)-{\left(A_{f}M\right)}_{i}h\left(t\right)-A_{f,i}f\left(t\right)+d_{i}\left(t\right)\;{\dot{d}}_{i}\left(t\right)=-\varsigma sign\left(e_{z,i}\left(t\right)\right)-\varphi e_{z,i}\left(t\right)\;\;\;i=1,2,..,p,$$

where ${\left(A_{f}C\right)}_{i}$, $A_{f,i}$, and ${\left(A_{f}M\right)}_{i}$ are the *i*th row of $A_{f}C$, $A_{f}$, and $A_{f}M$, respectively. By defining a new variable

(15) $$d_{0,i}\left(t\right)={\left(A_{f}C\right)}_{i}e_{1}\left(t\right)-{\left(A_{f}M\right)}_{i}h\left(t\right)-A_{f,i}f\left(t\right)+d_{i}\left(t\right)\;\;\;i=1,2,..,p,$$

the Equation (14) can be rewritten as

(16) $${\dot{e}}_{z,i}\left(t\right)=-\psi sign\left(e_{z,i}\left(t\right)\right){\left|e_{z,i}\left(t\right)\right|}^{1/2}-\chi e_{z,i}\left(t\right)+d_{0,i}\left(t\right)\;\;{\dot{d}}_{0,i}\left(t\right)=-\varsigma sign\left(e_{z,i}\left(t\right)\right)-\varphi e_{z,i}\left(t\right)+\phi_{i}\left(t\right)\;\;\;i=1,2,..,p,$$

where $\phi_{i}\left(t\right)={\left(A_{f}C\right)}_{i}{\dot{e}}_{1}\left(t\right)-{\left(A_{f}M\right)}_{i}\dot{h}\left(t\right)-A_{f,i}\dot{f}\left(t\right)$. Then

(17) $$\left\|\phi_{i}\left(t\right)\|<\|{\left(A_{f}C\right)}_{i}\|\|{\dot{e}}_{1}\left(t\right)\|+\|{\left(A_{f}M\right)}_{i}\|\|\dot{h}\left(t\right)\|+\|A_{f,i}\|\|\dot{f}\left(t\right)\right\|.$$

Since A is stable, the autonomous system associated with $e_{1}\left(t\right)$ is stable. Consequently, both $\left\|e_{1}\left(t\right)\right\|$ and $\left\|{\dot{e}}_{1}\left(t\right)\right\|$ are bounded. Provided $\left\|{\dot{h}}_{1}\left(t\right)\right\|$ and $\left\|{\dot{f}}_{1}\left(t\right)\right\|$ are bounded, then there exists a sufficiently large ε with which $\|\phi_{i}\left(t\right)\|<\varepsilon$ is satisfied. As discussed in [11], Equation (16) is a special case of the super-twisting structure from [12]. Choose the scalar gains from Equation (16) as

(18) $$\psi >2\sqrt{\varepsilon },\chi >0,\varsigma >\varepsilon ,\varphi >\frac{\chi^{2}\left(\psi^{3}+5/4\psi^{2}+5/2\left(\varsigma -\varepsilon \right)\right)}{\psi \left(\varsigma -\varepsilon \right)},$$

Consequently, from the results of [12], it can be proven that a sliding motion will take place and ${\dot{e}}_{z,i}\left(t\right)=e_{z,i}\left(t\right)=0$ in finite time.

Once the sliding surface is reached, the error dynamics in Equation (12) are given by

(19) $${\dot{e}}_{1}\left(t\right)=Ae_{1}\left(t\right)-Lh\left(t\right)\;0=A_{f}Ce_{1}\left(t\right)+I_{p}\nu_{eq}\left(t\right)-A_{f}Mh\left(t\right)-A_{f}f\left(t\right),$$

where the signal $\nu_{eq}\left(t\right)$ is the so-called equivalent output injection signal. As in [13], $\nu_{eq}\left(t\right)$ represents the averaged behavior of ν(t), which can be obtained from ν(t) by a low pass filter. Considering only the effects of f(t), i.e., assuming $A_{f}Mh\left(t\right)=0$, the sensor fault reconstruction can be obtained from

(20) $$\hat{f}\left(t\right)=A_{f}^{-1}\nu_{eq}\left(t\right).$$

## 4. Degraded Performance Tracking and Post-Flight Model Update

In the previous section, a second order sliding mode observer has been constructed for engine sensor fault diagnosis. Although the health parameters were considered during the analysis, the reconstruction signal was approximated by leaving out the effect of h(t). The sliding mode observer is able to obtain robust stability with a proper design of gains, but the pollution in $\hat{f}\left(t\right)$ caused by h(t) may generate misdiagnosis or false alarm in in-flight sensor fault diagnostics. In this section, the problem is investigated by constructing another sliding mode observer to update the degraded engine model post-flight. That is, the health parameters are estimated on-line in each flight, and then the baseline model for sensor diagnostics is updated post-flight by using the analyzed health tracking data, eventually in next flight the updated model will be used for on-board sensor fault diagnosis, instead of the non-degraded baseline model. In this section, the observer for tracking performance degradation is designed by the same methodology applied previously. The degraded engine model can be expressed as

(21) $$\dot{x}\left(t\right)=Ax\left(t\right)+Bu\left(t\right)+Lh\left(t\right)\;\;y\left(t\right)=Cx\left(t\right)+Du\left(t\right)+Mh\left(t\right).$$

Here, the output vector is partitioned as ${\left[y_{1}^{T},y_{2}^{T}\right]}^{T}$ such that $y_{1}={\left[N_{L},N_{H}\right]}^{T}$ and $y_{2}={\left[T_{25},P_{25},T_{3},P_{3},T_{495}\right]}^{T}$, the reformulated model becomes

(22) $$\dot{x}\left(t\right)=Ax\left(t\right)+Bu\left(t\right)+Lh\left(t\right)\;\{\begin{array}{c} y_{1}\left(t\right)=C_{1}x\left(t\right)+D_{1}u\left(t\right)+M_{1}h\left(t\right)\\ y_{2}\left(t\right)=C_{2}x\left(t\right)+D_{2}u\left(t\right)+M_{2}h\left(t\right) \end{array},$$

where $C_{1}$, $C_{2}$, $D_{1}$, $D_{2}$, $M_{1}$, and $M_{2}$ are coefficient matrices of appropriate dimension. Since $y_{1}=\;x$, then $C_{1}$ is an identity matrix, and $D_{1}$, $M_{1}$ are both zero matrices. Obviously, there is no useful information in measurement equation of $y_{1}$ for health tracking usage. Consider a filtered version of only $y_{2}$, which is

(23) $${\dot{z}}_{2}\left(t\right)=-A_{f^{\prime }}z_{2}\left(t\right)+A_{f^{\prime }}y_{2}\left(t\right),$$

where $-A_{f^{\prime }}\in {\mathbb{R}}^{\left(p-n\right)\times \left(p-n\right)}$ is a stable filter matrix. Similarly, substituting $z_{2}\left(t\right)$ for $y_{2}\left(t\right)$ in Equation (22), and combining x(t) and $z_{2}\left(t\right)$ to create $x_{a^{\prime }}\left(t\right)\in {\mathbb{R}}^{p}$, the augmented system is in the form of

(24) $$\left[\begin{array}{c} \dot{x}\left(t\right)\\ {\dot{z}}_{2}\left(t\right) \end{array}\right]=\underset{A_{a^{\prime }}}{\underset{︸}{\left[\begin{array}{cc} A & 0\\ A_{f^{\prime }}C_{2} & -A_{f^{\prime }} \end{array}\right]}}\underset{x_{a^{\prime }}\left(t\right)}{\underset{︸}{\left[\begin{array}{c} x\left(t\right)\\ z_{2}\left(t\right) \end{array}\right]}}+\underset{B_{a^{\prime }}}{\underset{︸}{\left[\begin{array}{c} B\\ A_{f^{\prime }}D_{2} \end{array}\right]}}u\left(t\right)+\underset{H_{a^{\prime }}}{\underset{︸}{\left[\begin{array}{c} L\\ A_{f^{\prime }}M_{2} \end{array}\right]}}h\left(t\right)\;y_{a^{\prime }}\left(t\right)=C_{a^{\prime }}x_{a^{\prime }}\left(t\right),$$

where $A_{a^{\prime }}\in {\mathbb{R}}^{p\times p}$, $B_{a^{\prime }}\in {\mathbb{R}}^{p\times m}$, $H_{a^{\prime }}\in {\mathbb{R}}^{p\times q}$, and $C_{a^{\prime }}=I_{p}$. Compared to Equation (6), the system in Equation (24) is reduced by 2 while the information necessary for health tracking is well reserved. The observer existence conditions discussed earlier are checked by $rank\left(C_{a^{\prime }}H_{a^{\prime }}\right)=rank\left(H_{a^{\prime }}\right)=q$, and

(25) $$rank\left(R_{a}\left(s\right)\right)=rank\left(\left[\begin{array}{cc} \begin{array}{cc} sI_{n}-A & 0\\ -A_{f^{\prime }}C_{2} & sI_{p-n}+A_{f^{\prime }} \end{array} & \begin{array}{c} L\\ A_{f^{\prime }}M_{2} \end{array}\\ I_{p} & 0 \end{array}\right]\right)\;=rank\left(I_{p}\right)+rank\left(\left[\begin{array}{c} L\\ A_{f^{\prime }}M_{2} \end{array}\right]\right)=p+q=2p,$$

which means the invariant zeros of $\left(A_{a^{\prime }},H_{a^{\prime }},C_{a^{\prime }}\right)$ are non-existant. With the conditions been checked, the same SOSMO design strategy for sensor fault can be used to fulfill degrading estimation based on system in Equation (24). With appropriate gains selected, the estimation of health parameters can be obtained from

(26) $$\hat{h}\left(t\right)=H_{a^{\prime }}^{-1}\nu_{eq}\left(t\right).$$

During flights, $\hat{h}\left(t\right)$ can be obtained on-line in real time. Since health degradations are assumed to be a long-time slow-evolving process, h(t) varies marginally in one flight. Therefore, define

(27) $$\hat{h}\left[k\right]=\frac{1}{T}\int_{0}^{T}\hat{h}\left(t\right)$$

as the estimating value of health parameters after the *k*th flight, where T is the flight cycle period. By ignoring the influence of degradation in the (*k +* 1)th flight, the on-board sensor fault diagnosis is modified to be based on the updated model

(28) $${\dot{x}}_{a}\left(t\right)=A_{a}x_{a}\left(t\right)+B_{a}u\left(t\right)+H_{a}\hat{h}\left[k\right]+F_{a}f\left(t\right)\;\;\hat{z}\left(t\right)=C_{a}{\hat{x}}_{a}\left(t\right).$$

Therefore, the observer for sensor reconstruction becomes

(29) $${\dot{\hat{x}}}_{a}\left(t\right)=A_{a}{\hat{x}}_{a}\left(t\right)+B_{a}u\left(t\right)+H_{a}\hat{h}\left[k\right]-G_{l}e_{z}\left(t\right)+G_{n}\nu \left(t\right)\;\;\hat{z}\left(t\right)=C_{a}{\hat{x}}_{a}\left(t\right).$$

By subtracting Equation (29) from Equation (24), the error system becomes

(30) $$\dot{e}\left(t\right)=A_{a}e\left(t\right)-G_{l}e_{z}\left(t\right)+G_{n}\nu \left(t\right)-F_{a}f\left(t\right)+\;H_{a}\left(\hat{h}\left[k\right]-h\left(t\right)\right).$$

**Figure 2:**
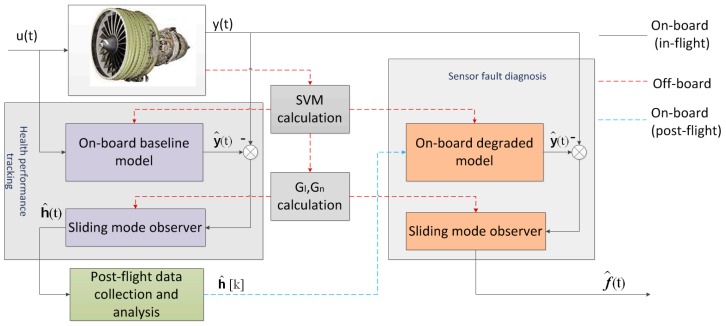
The overall architecture of the integrated engine sensor fault diagnostic system.

Since in the (*k +* 1)th flight $h\left(t\right)\approx \hat{h}\left[k\right]$, the effect of performance degradation is eliminated from the error system and the following deductions. Therefore, the sensor diagnostic system maintains its effectiveness even in performance degradation circumstances. The overall sensor fault diagnostic scheme can be summarized as: one SMO is built for on-board health performance estimation, then the calculation of $\hat{h}\left[k\right]$ is done after each flight to update the current degraded baseline model, which is used for the on-board sensor fault diagnostic algorithm based on another SMO. An overview of the model updating and sensor fault diagnostic architecture is presented in Figure 2.

From the observer form in Equation (29), it can be seen that although the degraded engine model is changed constantly with updated $\hat{h}\left[k\right]$, the observer gains do not need adjustments. It is a significant advantage compared to the traditional KF-bank-based methods in [3,4], which demands the diagnostic algorithm to be redesigned once the degraded model is updated, and the redesigned algorithm to be uploaded to the on-board computer. The frequency of the periodic update in their schemes is hard to decide: shortening the update period means decreasing the model errors, but it will inevitably increase the labor, material, and cost consumption. Whereas in the proposed scheme, the updating process being implemented each flight can be handled just with an on-board computer, and human effort which is necessary in [3,4] to adjust the algorithms is avoided. Thus the update process can be executed after each flight, which provides a more accurate degraded model for sensor fault reconstruction.

## 5. Simulation and Performance Evaluation

In this section, simulation results and performance evaluations of the proposed sensor fault diagnostic system for a commercial aircraft engine are presented. Although the algorithms are based on the state-space model, the verification process will be conducted via a nonlinear component-level model (CLM) of a twin-spool turbofan engine, which is a simulation platform as a representative of a commercial turbofan engine with highly fidelity. The developed CLM was described in [14], and it has been validated against testing data extracted from the real engine. The CLM consists of a set of individual components, such as compressors, combustors, and turbines, etc. Each component contains mathematical equations, maps, and tables describing the thermodynamic relationships between various variables, and requires a number of inputs and generates one or more variables. The thermodynamic parameters in cross sections of each component—such as the total temperature, total pressure, efficiency, and flow capacity—can be calculated as in [15]. The steady state simulation of CLM is based on mass flow balance and power balance equations, while the transient simulation, initialed by steady state calculation, follows mass flow balance and rotor dynamics equations. The Newton-Raphson method is employed to solve the nonlinear expressions both in steady state case and transient dynamics. Iterative solution of nonlinear equations in each step stops once the iteration number reaches 10, or the iteration error is less than 0.01. The CLM is written using C language and packaged with dynamic link library (DLL) for use in the MATLAB environment [15,16]. The health parameters are modeled and health degrading injection is available in the CLM. Sensor dynamics are ignored in the simulations, with the assumption that they have high enough bandwidth. 

The CLM is simulated at the reference flight condition and at a nominal cruise power setting, with H=10.7 km, $M_{a}=0.78$, and $W_{f}=0.36\;\mathrm{kg}/s$. To represent real working condition, the white Gaussian measurement noise and process noise are introduced with standard deviations (percentage of the nominal value) $\sigma_{noise,m}=0.0015$ and $\sigma_{noise,p}=0.0005$, respectively. The magnitude of noises is determined by practical experience and previously published data [14].

**Figure 3:**
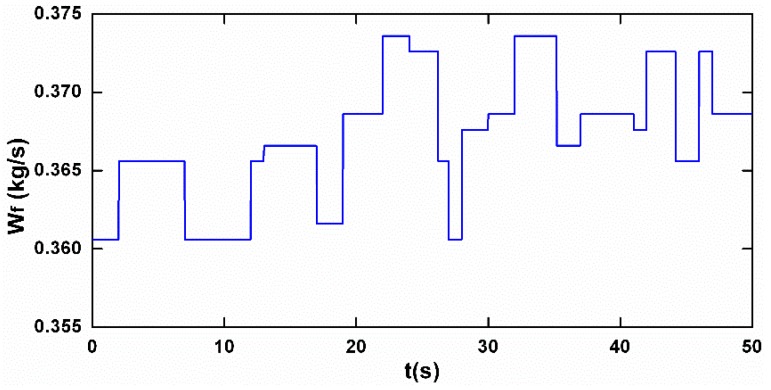
Fuel flow rate schedule.

One issue should be addressed that, in the real flight environment, the ambient condition continually changes even in the cruise phase, such as the inlet temperature and pressure. Generally, the target of the steady control scheme for an aero-engine is a constant value of $N_{L}$, thus the change of the ambient condition means the fuel flow is regulated real-time to keep $N_{L}$ invariant. The sensor fault diagnostic system should be developed to be robust enough to handle $W_{f}$ movements. In view of this, in the following simulations, a continuously varying $W_{f}$ is employed to present real cruise conditions, as shown in Figure 3, and the proposed system is evaluated for its diagnostic capabilities during $W_{f}$ transient.

**Figure 4:**
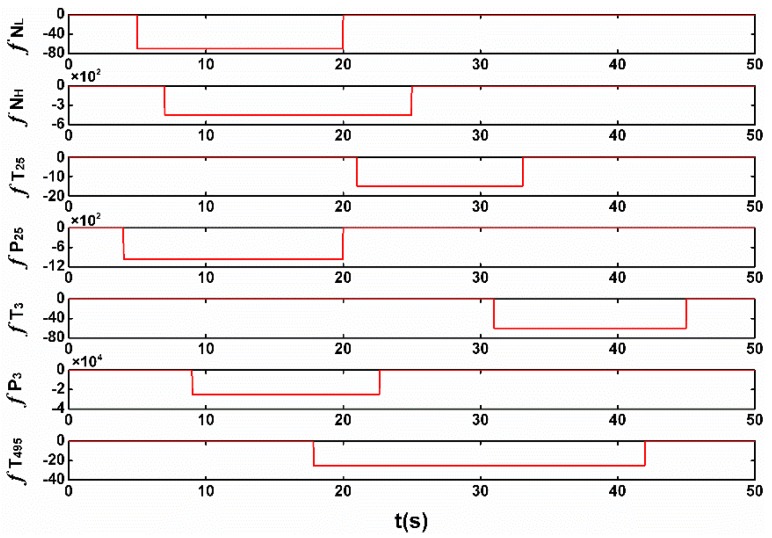
The injected sensor faults.

The sensor faults being dealt with in this paper are “soft” failures, which are defined as inconsistencies between true and measured sensor values that are relatively small in magnitude. Oppositely, “hard” failures are large in magnitude and thus more readily detectable. Sensor faults are in different forms such as a step or a drift, and in the simulations step faults are applied, which are considered “harsher” to the system, and are convenient to evaluate observer dynamic performance. To examine the proposed scheme, an extreme case in which faults with different magnitude occur in the entire seven sensors during 50 s in one flight is carried out, as shown in Figure 4. The nominal value of the measurements at the cruise and the fault magnitude in percentage are shown in Table 2.

**Figure 5:**
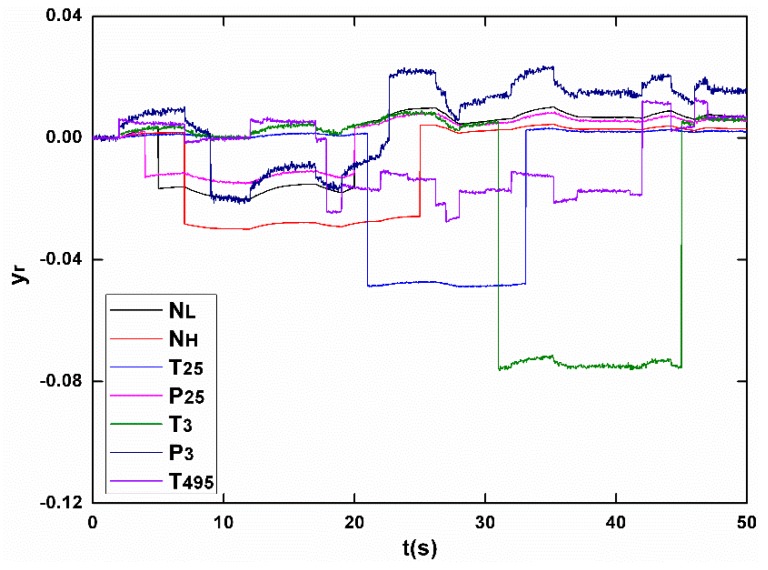
The normalized measurements.

**Figure 6:**
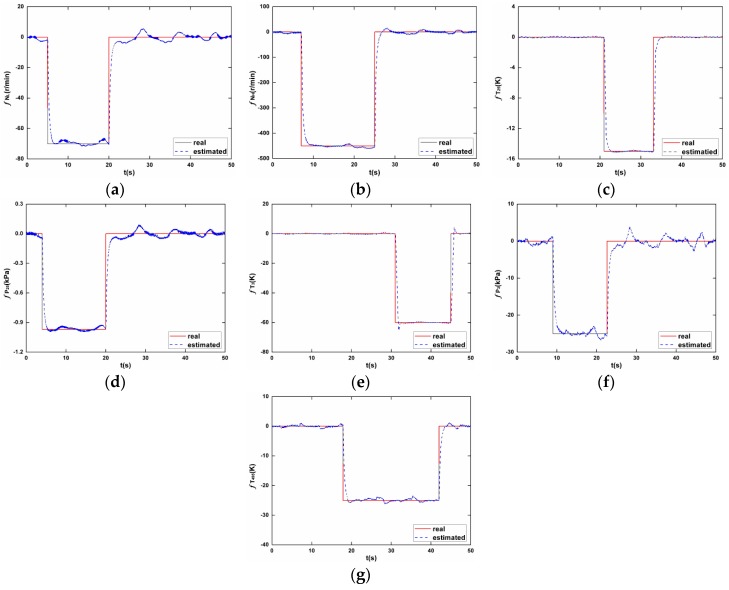
The sensor fault reconstruction results. (**a**) $N_{L}$; (**b**) $N_{H}$; (**c**) $T_{25}$; (**d**)$\;P_{25}$; (**e**) $T_{3}$; (**f**) $P_{3}$; (**g**) $T_{495}$.

The capability of the proposed scheme is first verified in non-degraded condition. Figure 5 shows the output variations in which the output are normalized as

(31) $$y_{r}=\left(y-y_{ds}\right)/y_{ds},$$

where $y_{ds}$ represents the nominal value of measurements at cruise operating point. The joint influence by fuel flow transient and fault injections makes it a tough task to identify each sensor fault correctly. However, in the proposed sensor fault diagnostic scheme, the sliding mode observer is designed to be robust enough to endure model mismatches caused by $W_{f}$ changes, while being able to reconstruct sensor faults with satisfactory accuracy. Figure 6 shows the diagnosing results, in which the seven concurrently occurring faults have been faithfully detected and reconstructed in separate sensors. Compared to the traditional FDI scheme based on KF-bank concept, the proposed scheme is able to not only detect and isolate faults, but the exact type and magnitude of faults are also obtained.

**Figure 7:**
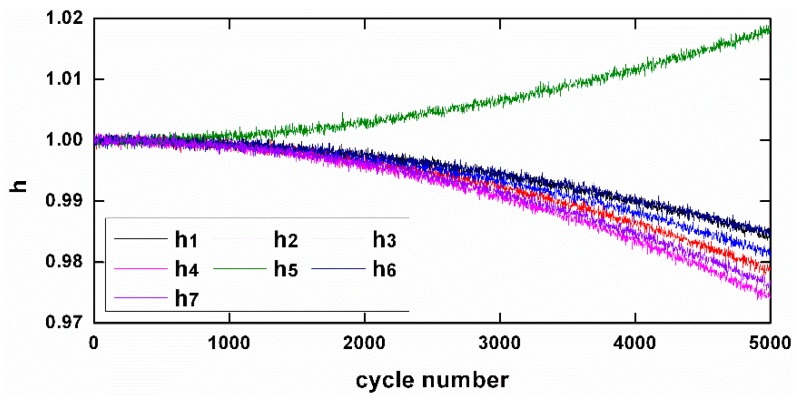
The health degradations during 5000 flight cycles.

**Figure 8:**
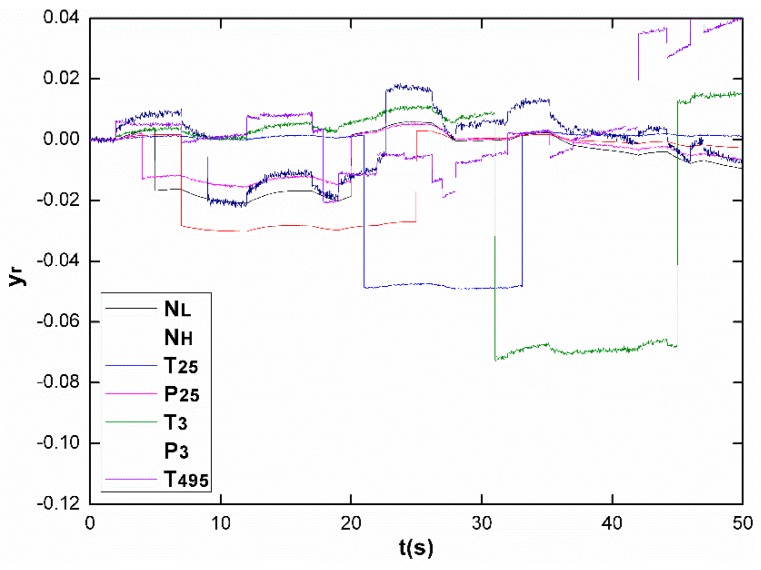
The normalized measurements.

**Figure 9:**
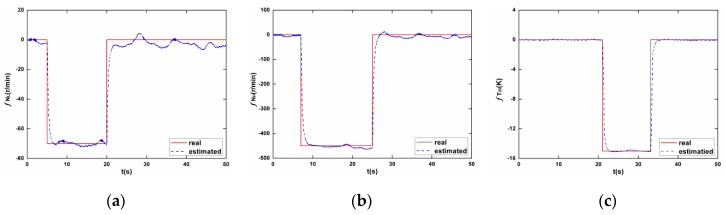

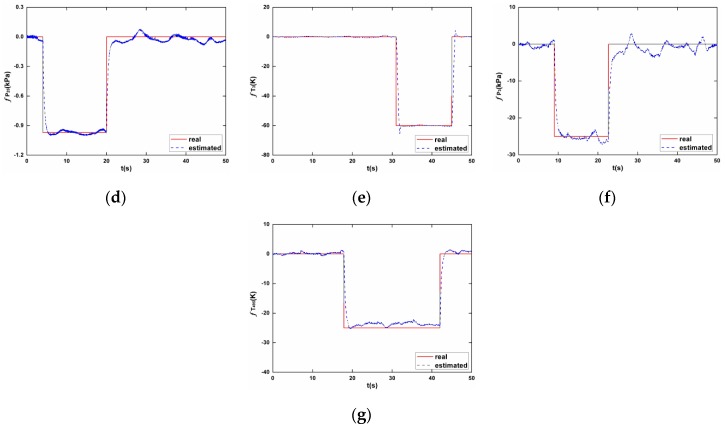
The sensor fault reconstruction results. (**a**) $N_{L}$; (**b**) $N_{H}$; (**c**) $T_{25}$; (**d**)$\;P_{25}$; (**e**) $T_{3}$; (**f**) $P_{3}$; (**g**) $T_{495}$.

Next, the proposed system is simulated to be employed in the course of an engine life with slow aging process. As an engine inevitably degrades overtime, it is necessary to evaluate the effectiveness of the sensor fault diagnostic system in such a real working condition. Figure 7 depicts the slow aging process reflected by health parameters, which slowly drift away from their nominal values in 5000 flight cycles. Assuming that the same sensor faults in Figure 3 occur during the 4501th flight cycle, Figure 8 shows the normalized outputs. Obviously the “information” in outputs for sensor fault reconstruction is corrupted by degradations. However, with another observer designed above for tracking degradation, before the 4501th flight an estimation value of health parameters representing the condition in the 4500th flight ($\hat{h}\left[4500\right]$) has been calculated. $\hat{h}\left[4500\right]$ is then employed to update the engine model used for sensor on-board diagnosis in the 4501th flight. With the update strategy, the sensor fault diagnosing results are shown in Figure 9. Due to the degraded model update, the sensor fault reconstructions are slightly affected. The accuracy of reconstruction is assessed in terms of the rooted square mean error (RSME). Table 3 shows the statistics reflecting the RSME results of seven sensor fault reconstruction signals in both non-degrading and degrading cases. The results consistently imply the ascendency of the proposed scheme in accuracy and robustness to degradation.

## 6. Conclusions

In this paper, the second-order sliding mode observers have been developed for building an integrated sensor fault diagnostic system of a commercial aircraft engine. With two observers separately designed for health performance tracking and sensor fault reconstruction, the proposed approach is capable of quickly detecting fault occurrence and accurately reshaping the fault profiles despite the presence of degradation. The post-flight degraded model update does not require observers to be regulated or redesigned. Being time and cost-saving, it implies great potential in real applications. The proposed scheme validated with nonlinear CLM reveals promising results in terms of the fast and accurate sensor fault reconstructions, both in non-degraded and degraded cases.

## Figures and Tables

**Table 1:** The notations and their descriptions.

Notation	Description
*H*	Height
*M*_a_	Mach number
*N*_L_	Low pressure rotor speed
*N*_H_	High pressure rotor speed
*h*	Health parameter vector
*h*_1_	LPC efficiency
*h*_2_	HPC efficiency
*h*_3_	HPT efficiency
*h*_4_	LPT efficiency
*h*_5_	LPC flow capacity
*h*_6_	HPC flow capacity
*h*_7_	HPT flow capacity
*h*_8_	LPT flow capacity
*W*_f_	Fuel flow rate
*P*_25_	HPC inlet pressure
*T*_25_	HPC inlet temperature
*P*_3_	Combustor inlet pressure
*T*_3_	Combustor inlet temperature
*T*_495_	Exhaust gas temperature

**Table 2:** The nominal value and the fault magnitude of sensors at cruise.

Measurement	Nominal Value	Fault Magnitude
$N_{L}$	3484 RPM	−2%
$N_{H}$	15,044 RPM	−3%
$T_{25}$	298 K	−5%
$P_{25}$	64,990 Pa	−1.5%
$T_{3}$	747 K	−8%
$P_{3}$	1,242,145 Pa	−2%
$T_{495}$	936 K	−2.5%

**Table 3:** The RMSE results of the sensor fault reconstruction (%).

	Non-Degrading Case	Degrading Case
$N_{L}$	0.197	0.208
$N_{H}$	0.294	0.298
$T_{25}$	0.515	0.516
$P_{25}$	0.143	0.146
$T_{3}$	0.895	0.895
$P_{3}$	0.219	0.236
$T_{495}$	0.241	0.252
